# Oromandibular dystonia: a serious side effect of capecitabine

**DOI:** 10.1186/s12885-015-1132-1

**Published:** 2015-03-11

**Authors:** Melanie JM van Pelt-Sprangers, Eric CT Geijteman, Jelmer Alsma, Ingrid A Boere, Ron HJ Mathijssen, Stephanie CE Schuit

**Affiliations:** 1 Department of Internal Medicine, Erasmus University Medical Center, P.O. Box 2040,, 3000 CA Rotterdam, the Netherlands; 2 Department of Medical Oncology, Erasmus MC Cancer Institute, Erasmus University Medical Center, P.O. Box 2040,, 3000, CA Rotterdam, the Netherlands; 3 Department of Emergency Medicine, Erasmus University Medical Center, P.O. Box 2040,, 3000, CA Rotterdam, the Netherlands

**Keywords:** Capecitabine, Side effect, Oromandibular dystonia, Anticholinergic drugs

## Abstract

**Background:**

Capecitabine has activity against several types of cancer. In 10-15% of patients treated with capecitabine, treatment is discontinued because of serious adverse reactions, mostly within the first weeks of treatment.

**Case presentation:**

A 56 year-old female patient presented at the emergency department after ten days of chemotherapy with progressive airway obstruction and complaints of numbness of the tongue. She also had difficulty swallowing and was unable to speak. Laboratory findings were completely normal and no co-medication was used, in particular no dopamine antagonists.

A diagnosis of oromandibular dystonia due to capecitabine use was made. After the anticholinergic drug biperiden (Akineton) was given intravenously, complaints disappeared within twenty minutes. Due to an early discontinuation of biperiden, however, the symptoms of oromandibular dystonia recurred. Again, she was successfully treated with an anticholinergic drug. Capecitabine was permanently discontinued. Three days after the initial presentation the anticholinergic drug was stopped after which symptoms did not reappear.

**Conclusion:**

The case highlights the need for awareness that capecitabine may potentially lead to severe life-threatening complaints of oromandibular dystonia. We hypothesize that capecitabine passed the blood brain barrier which led to a disruption within the basal ganglia in this case. Prompt treatment with an anticholinergic drug and cessation of capecitabine in the patient case led to disappearance of complaints.

## Background

Capecitabine has proven activity against several types of cancers, including those of the gastro-intestinal tract, and breast. It is an oral prodrug that is converted to its active metabolite 5-fluorouracil (5-FU). Together with tegafur, which is another oral prodrug, and 5-FU itself, capecitabine belongs to the group of fluoropyrimidines. Common dose-limiting systemic toxicities are hand-foot syndrome and diarrhoea. Because the final converting step of capecitabine to 5-FU is believed to take mainly place in the tumour, side effects of capecitabine are presumably less than with 5-FU [1]. However, in 10%-15% of the patients treated with capecitabine treatment is discontinued because of adverse reactions [2,3]. Most of these discontinuations are necessary within the first weeks of treatment [3]. Here, we present a rare but serious case of severe oromandibular dystonia shortly after starting capecitabine.

## Case presentation

A 56 year-old Caucasian woman who was diagnosed with a T3N2M0 rectal cancer, underwent neoadjuvant chemoradiotherapy with capecitabine. She presented at the emergency department after ten days of treatment with capecitabine (1,500 mg BID).

During the six days prior to presentation, she developed progressive symptoms of cramps between her shoulders, a tingling feeling in both arms and numbness of the tongue. She also had difficulty swallowing and was unable to speak. Symptoms were intermittent, but progressive during these six days, leading to progressive airway obstruction at the day of presentation. She did not have any features of typical capecitabine toxicity like hand-foot syndrome or mucositis, nor were there any other focal neurological signs. Laboratory findings were completely normal and no co-medication was used, in particular no dopamine antagonists.

A diagnosis of oromandibular dystonia due to capecitabine use was made, and the drug was stopped immediately. The anticholinergic drug biperiden (Akineton) 10 mg was given intravenously, after which speaking and tongue movements improved within twenty minutes.

Unfortunately, despite prescription of biperiden 5 mg, it was inadvertently not given, and twelve hours after presentation the symptoms of oromandibular dystonia with difficult speaking and tongue numbness recurred in the same intensity as at presentation. Again, she was successfully treated with biperiden intravenously.

After the patient was able to swallow again, a switch to an oral anticholinergic (1 mg of trihexyfenidyl (Artane) once daily) was made during three days. Symptoms did not reappear and the patient was successfully discharged from the hospital. Pharmacogenetic counseling, performed after the side effect appeared, showed a DPYD *1/*1 genotype, which corresponds to a normal 5-FU drug metabolism.

## Discussion

We report an extremely rare, but clinically highly relevant, case of capecitabine induced oromandibular dystonia, leading to progressive airway obstruction without any other associated neurological signs. We concluded that this was due to capecitabine use, because after discontinuing bipiriden treatment for the acute dystonia, symptoms reappeared, which may be seen as a rechallenge phenomenon and after cessation of capecitabine she recovered completely and complaints never recurred. Moreover, the assessment by the Naranjo causality scale – a method for estimating the probability of adverse drug reactions – [4] showed that the adverse drug reaction was definitely related to the drug (see Table 1). Furthermore, other – rare – causes of oromandibular dystonia, such as the use of metoclopramide, were excluded.

**Table 1:** **The Naranjo Causality Scale**

	Yes	No	Do not know	Score case
1. Are there previous conclusive reports on this reaction?	+1	0	0	+1
2. Did the adverse event occur after the suspected drug was administered?	+2	−1	0	+2
3. Did the adverse reaction improve when the drug was discontinued or a specific antagonist was administered?	+1	0	0	+1
4. Did the adverse reaction reappear when the drugs was readministered? (‘rechallenge’)	+2	−1	0	+2*
5. Are there alternative causes (other than the drug) that could have on their own caused the reaction?	−1	+2	0	+2
6. Did the reaction reappear when a placebo was given?	−1	+1	0	0
7. Was the blood detected in any body fluid in toxic concentrations?	+1	0	0	0
8. Was the reaction more severe when the dose was increased, or less severe when the dose was decreased?	+1	0	0	0
9. Did the patient have a similar reaction to the same or similar drugs in any previous exposure?	+1	0	0	0
10. Was the adverse event confirmed by any objective evidence?	+1	0	0	+1
Scoring				
≥9 = definite ADR				
5-8 = probable ADR				
1-4 = possible ADR				
0 = doubtful ADR				

*We answered this question with ‘yes’. Although capecitabine was not readministered, biperiden was discontinued. By doing so, the patient could be re-exposed to the side-effects of capecitabine and its metabolites which in all probability had not been completely eliminated.

Our case is the first reported case of oromandibular dystonia due to capecitabine in Caucasians. In an earlier described case a Chinese male patient developed oromandibular dystonia nine days after swallowing capecitabine which resolved spontaneously after three days [5]. However, a feeding tube had to be inserted because he had difficulty swallowing during these days. To the best of our knowledge oromandibular dystonia is never reported after the administration of other forms of 5-FU. Focal dystonia, however, caused by 5-FU has been reported in one earlier case series including three cases [6]. Due to the serious nature of this adverse event, although rare, we are convinced that physicians should be aware of this.

Fluoropyrimidine toxicity can be predicted by certain pharmacogenetic markers [7]. A main marker is dihydropyrimidine dehydrogenase (DPD) [8]. DPD is the rate-limiting enzyme for fluoropyrimidine catabolism that eliminates >80% of administered 5-fluorouracil [9]. The most predominant polymorphism associated with DPD deficiency is DPYD*2A [10], which was excluded in our case. Therefore it is unlikely that the side-effect reported here is due to DPD deficiency [8].

It is thought that abnormalities in neurotransmitters resulting in disturbed firing patterns of the basal ganglia are involved in the case of oromandibular dystonia [11]. The pathophysiological mechanism by which oromandibular dystonia occurs upon capecitabine intake is unclear. One plausible explanation is that capecitabine may pass through the blood brain barrier [5], which may lead to a disruption within the basal ganglia, the centre for movement control. Such disruption is seen with other types of dystonia and other causes of oromandibular dystonia [12-14]. The rapid improvement after anticholinergic drugs, which is the first choice of systemic treatment in these other types of dystonia [15], underlines a similar pathogenesis. That capecitabine may pass through the blood brain barrier is affirmed by its activity in patients with brain metastases from breast cancer [16], together with other reported capecitabine central nervous system toxicity, such as cerebellar toxicity [17].

## Conclusion

Our case highlights the need for awareness that capecitabine may potentially lead to severe life-threatening complaints of oromandibular dystonia. More research is needed to clarify the pathogenesis of oromandibular dystonia in case of capecitabine intake. When faced with a patient with oromandibular dystonia we suggest prompt treatment with an anticholinergic drug, such as bipiriden, and cessation of capecitabine.

## Consent

Written informed consent was obtained from the patient for publication of the Case Report. A copy of the written consent is available for review by the Editor of this journal.
